# Identification of mucin degraders of the human gut microbiota

**DOI:** 10.1038/s41598-021-90553-4

**Published:** 2021-05-27

**Authors:** Stefano Raimondi, Eliana Musmeci, Francesco Candeliere, Alberto Amaretti, Maddalena Rossi

**Affiliations:** 1 Department of Life Sciences, University of Modena and Reggio Emilia, 41125 Modena, Italy; 2 Biogest-Siteia, University of Modena and Reggio Emilia, 42122 Reggio Emilia, Italy

**Keywords:** Microbiology, Bacteria, Microbial communities

## Abstract

Mucins are large glycoproteins consisting of approximately 80% of hetero-oligosaccharides. Gut mucin degraders of healthy subjects were investigated, through a culture dependent and independent approach. The faeces of five healthy adults were subjected to three steps of anaerobic enrichment in a medium with sole mucins as carbon and nitrogen sources. The bacterial community was compared before and after the enrichment by 16S rRNA gene profiling. Bacteria capable of fermenting sugars, such as *Anaerotruncus*, *Holdemania,* and Enterococcaceae likely took advantage of the carbohydrate chains. *Escherichia coli* and Enterobacteriaceae, Peptococcales, the Coriobacteriale *Eggerthella*, and a variety of Clostridia such as Oscillospiraceae, *Anaerotruncus*, and *Lachnoclostridium*, significantly increased and likely participated to the degradation of the protein backbone of mucin. The affinity of *E. coli* and Enterobacteriaceae for mucin may facilitate the access to the gut mucosa, promoting gut barrier damage and triggering systemic inflammatory responses. Only three species of strict anaerobes able to grow on mucin were isolated from the enrichments of five different microbiota: *Clostridium disporicum*, *Clostridium tertium*, and *Paraclostridium benzoelyticum*. The limited number of species isolated confirms that in the gut the degradation of these glycoproteins results from cooperation and cross-feeding among several species exhibiting different metabolic capabilities.

## Introduction

Mucus is a complex gel barrier that covers the wet epithelial surfaces throughout the body, including the gastrointestinal tract, and offers protection against exogenous and endogenous aggressive agents^1,2^. It exerts a variety of functions such as lubrification, hydration, chemical protection, sensing, nutrient reservoir, and barrier against pathogen invasion. A continuous turnover, consisting in a dynamic balance between biosynthesis, secretion, and degradation of its structural components, is crucial for these functions^3^.

Mucins are major component of mucus, made up by glycoproteins with high level of glycosylation. Residues of galactose, N-acetylglucosamine (GlcNAc), N-acetylgalactosamine (GalNAc), fucose, and sialic acid, with relatively small amounts of mannose, constitute the oligosaccharides which represent approx. 80% of mucin mass^4^. The protein core is mainly organized in tandem repeated regions rich in serine, threonine, and proline. Serine and threonine are the site where an O-glycosidic bound links the protein core to GalNAc, the first moiety of the glycan chain^5,6^.

Among all the sites of human body inhabited by a resident microbial community, the colon hosts the most complex and concentrated microbiota^7^. In this site, mucins behave as decoy molecules that avoid the interaction of bacterial adhesins with receptors of the colonic epithelium. In fact, the gut lumen is coated by a two-layered mucus system, with an inner dense stratus firmly attached to the epithelium and an outer one, looser and unattached^8^. The inner layer is thick and stratified and prevents gut bacteria from reaching the epithelial cell surface. It is progressively converted into a laxer and expanded coating through the lytic action of proteases and glycosidases of both the host and the commensal bacteria. The outer mucus layer is the colonization site of the resident commensal microbiota, which differs from that of the digesta-associated and fecal content in terms of relative abundance of the different taxa^9^.

Mucus layers are the frontline of the interaction between the host and the gut bacteria, thus a balanced and symbiotic relationship which benefits both the former and the latter greatly relies on mucus structure^2^. Mucin plays a pivotal role in the selection of bacteria colonizing the mucus by supplying carbon and nitrogen substrates and exposing O-glycan chains that serve as attachment sites for colonization^4,8^. In turn, commensal bacteria feeding on and adhering to mucin limit the penetration of pathogens in the outer layer of the mucus^10^. These bacteria tightly interact with the host and modulate mucin gene expression, glycosylation, and secretion, thus affecting mucus and epithelial homeostasis^3,11^.

This study aimed to investigate mucin degraders of the gut, to unveil how mucins influence selection of resident bacteria and how specific bacteria shape the mucus layer. A medium containing mucin as sole carbon and nitrogen source (hereinafter referred to as MM medium) was utilized in strictly anaerobic enrichment cultures of human gut microbiota, inoculated with fresh feces of 5 healthy adults. The taxa that took advantage of mucins as growth substrate were identified with a 16S rRNA gene metataxonomic analysis, comparing the bacterial community of the fecal slurries (hereinafter referred to as FS) and the cultures after three enrichment steps (hereinafter referred to as EC). The bacteria able to grow using mucin as sole carbon and nitrogen source were isolated and taxonomically characterized from EC samples.

## Results

### Cultivable bacteria enriched on mucin

Intestinal mucin degrading bacteria were enumerated and isolated from EC samples, i.e. after three enrichment steps in MM broth. To discriminate anaerobic mucin degraders from mucinolytic Enterobacteriaceae, the enumeration was carried out with MM plates in anaerobiosis and MacConkey plates in aerobiosis (Table 1), and the isolates from MM plates were confirmed to be strict anaerobes. Strictly anaerobic mucin degraders were found in the magnitude of 10^8^ cfu/mL (mean ± SD = 8.3 ± 0.3 Log_10_ cfu/mL), significantly more abundant (P < 0.05) than mucinolytic Enterobacteriaceae, that ranged between 10^7^ and 10^8^ cfu/mL (mean ± SD = 7.6 ± 0.4 Log_10_ cfu/mL). The viable counts of both anaerobic mucin degraders and mucinolytic Enterobacteriaceae did not differ among the EC samples (ANOVA, P > 0.05).

**Table 1:** Cultivable mucin degraders in EC samples.The viable counts of anaerobic mucinolytic bacteria and Enterobacteriaceae, respectively counted onto plates of MM medium and MacConkey, and the taxonomic attribution of the predominant anaerobic is reported.

Sample	Anaerobic mucinolytic bacteria Log (cfu/mL)	Enterobacteriaceae Log (cfu/mL)	Isolated strains		Ref_seq ID
EC 1	7.8 ± 0.9	7.2 ± 0.5	*Clostridium disporicum*	WC0700	NR_026491.1
			*Clostridium disporicum*	WC0701	NR_026491.1
EC 2	8.4 ± 0.7	7.3 ± 0.4	*Clostridium disporicum*	WC0702	NR_026491.1
			*Clostridium disporicum*	WC0703	NR_026491.1
EC 3	8.5 ± 0.5	7.9 ± 0.5	*Clostridium disporicum*	WC0704	NR_026491.1
EC 4	8.3 ± 0.6	7.6 ± 0.6	*Paraclostridium benzoelyticum*	WC0705	NR_148815.1
			*Clostridium disporicum*	WC0706	NR_026491.1
EC 5	8.4 ± 0.5	8.0 ± 0.3	*Paraclostridium benzoelyticum*	WC0707	NR_148815.1
			*Paraclostridium benzoelyticum*	WC0708	NR_148815.1
			*Clostridium tertium*	WC0709	NR_113325.1

Counts are reported as mean Log (cfu/mL) ± standard deviation, n = 3. For both the media, no significant difference was observed among samples (ANOVA, P > 0.05). Cultivable anaerobic mucinolytic bacteria were significantly more numerous than Enterobacteriaceae (paired samples t-test, P < 0.05). Taxonomic attribution was obtained by alignment of the V1–V4 regions of 16S rRNA gene with the NCBI refseq_rna database using BLAST. The accession number of best hits and the sequence identity (%) obtained with MUSCLE are reported.

Based on RAPD-PCR fingerprinting, the anaerobic isolates clustered in 1–3 distinct biotypes *per* sample, for a total of 10 strains (Table 1). The partial sequences of 16S rRNA genes showed identity > 98.6% to the following species: *Clostridium disporicum* (6 biotypes), *Clostridium tertium* (1), and *Paraclostridium benzoelyticum* (3). These strains were not able to grow in MM plates without mucin.

### Microbiota of enrichment cultures grown on mucin

The metataxonomic survey of 16S rRNA gene in FS and EC samples yielded a total 415,051 sequences (31,153–55,162 *per* sample). The reads were dereplicated into 522 ASVs hitting a reference sequence in Silva database, and collapsed at the 7th level of taxonomic annotation into 150 OTUs (Supplementary Table 1).

**Figure 1:**
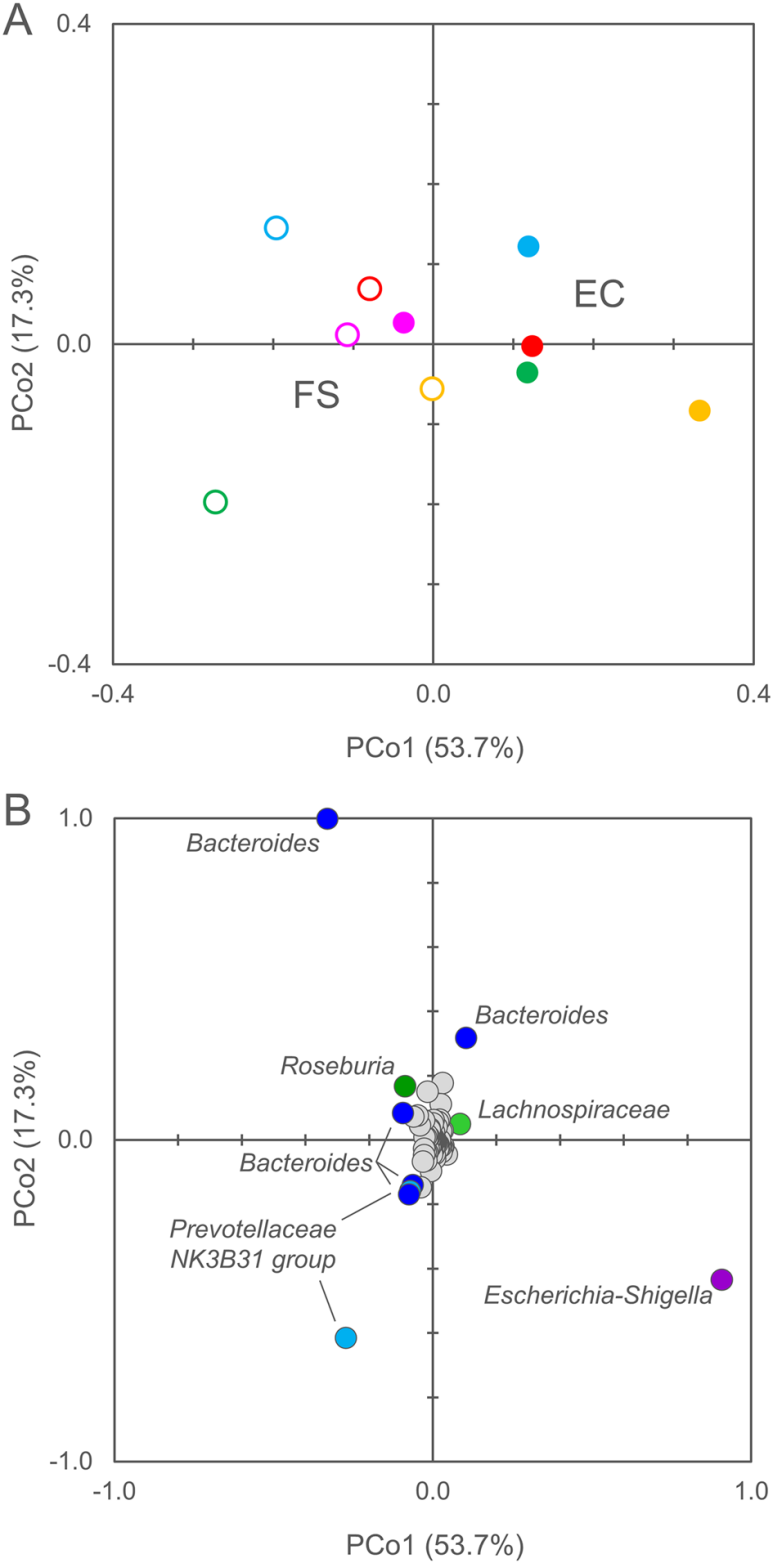
Beta diversity analysis of the microbiota in FS and EC samples. (**A**) PCo1-PCo2 visualization of distances computed with Weighted Unifrac, that considers phylogenetic relationships among bacteria. Symbols: FS, empty circle; EC, full circle; different colors correspond to different subjects (1, fuchsia; 2, cyan; 3, green; 4, red; 5, yellow). (**B**) PCo1-PCo2 visualization of the contribution of each ASV. The 10 ASVs exerting the highest effect on PCo1 are labelled and colored according to their phylum (Firmicutes, green; Bacteroidetes, blue; Proteobacteria, purple).

The analysis of microbiota alpha-diversity revealed that the richness (Chao1) and the evenness (Shannon and Pielou) tended to decrease with the enrichment but did not reach statistical significance (P > 0.05) (Supplementary Fig. 1). According to Weighted UniFrac computation of beta-diversity, FS and EC samples clustered in distinct groups (ANOSIM, PERMANOVA, P < 0.05) in a PCoA plot, with the former laying at lower values of PCo1 than the latter (Fig. 1A). *Escherichia-Shigella* presented the most positive contribution to PCo1, followed by ASVs of *Bacteroides* and of Lachnospiraceae, while other ASVs of *Bacteroides*, Prevotellaceae NK3B31, and *Roseburia* negatively weighted (Fig. 1B). Unweighted UniFrac yielded more dispersed groups (ANOSIM, PERMANOVA, P > 0.05), with EC samples laying at lower PCo2 compared to the corresponding FS ones (Supplementary Fig. 2A). The ASVs that negatively contributed to PCo2 included *Clostridium *sensu stricto* I* and other *Bacteroides*, while Prevotellaceae NK3B31 and *Eubacterium coprostanoligenes* were positive contributors (Supplementary Fig. 2B). Weighted UniFrac computation takes into account abundances of taxa, unlike Unweighted Unifrac that considers the mere presence or absence. Thus, the taxa that emerged from the former presented relevant changes in terms of relative abundance, while those that emerged from the latter likely appeared or disappeared over the enrichment steps.

**Figure 2:**
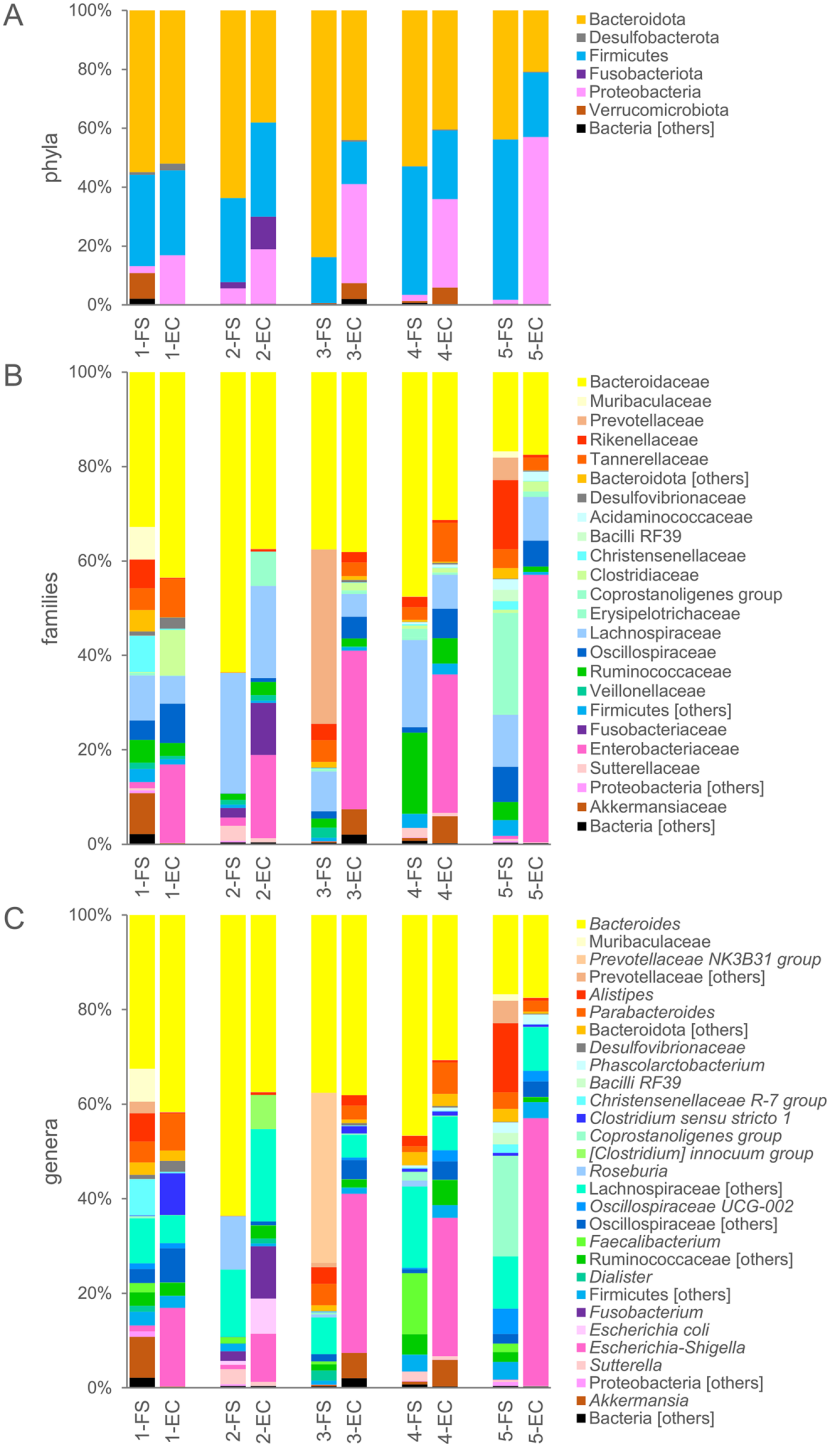
Stacked bar-plot representation of microbiota composition before and after the enrichment in MM, with taxonomic features collapsed at the level of phyla (**A**), families (**B**), and genera (**C**). The taxa that remained unclassified at the deeper level or that never occurred with abundance higher than 0.5% are grouped and marked with “[others]”.

**Figure 3:**
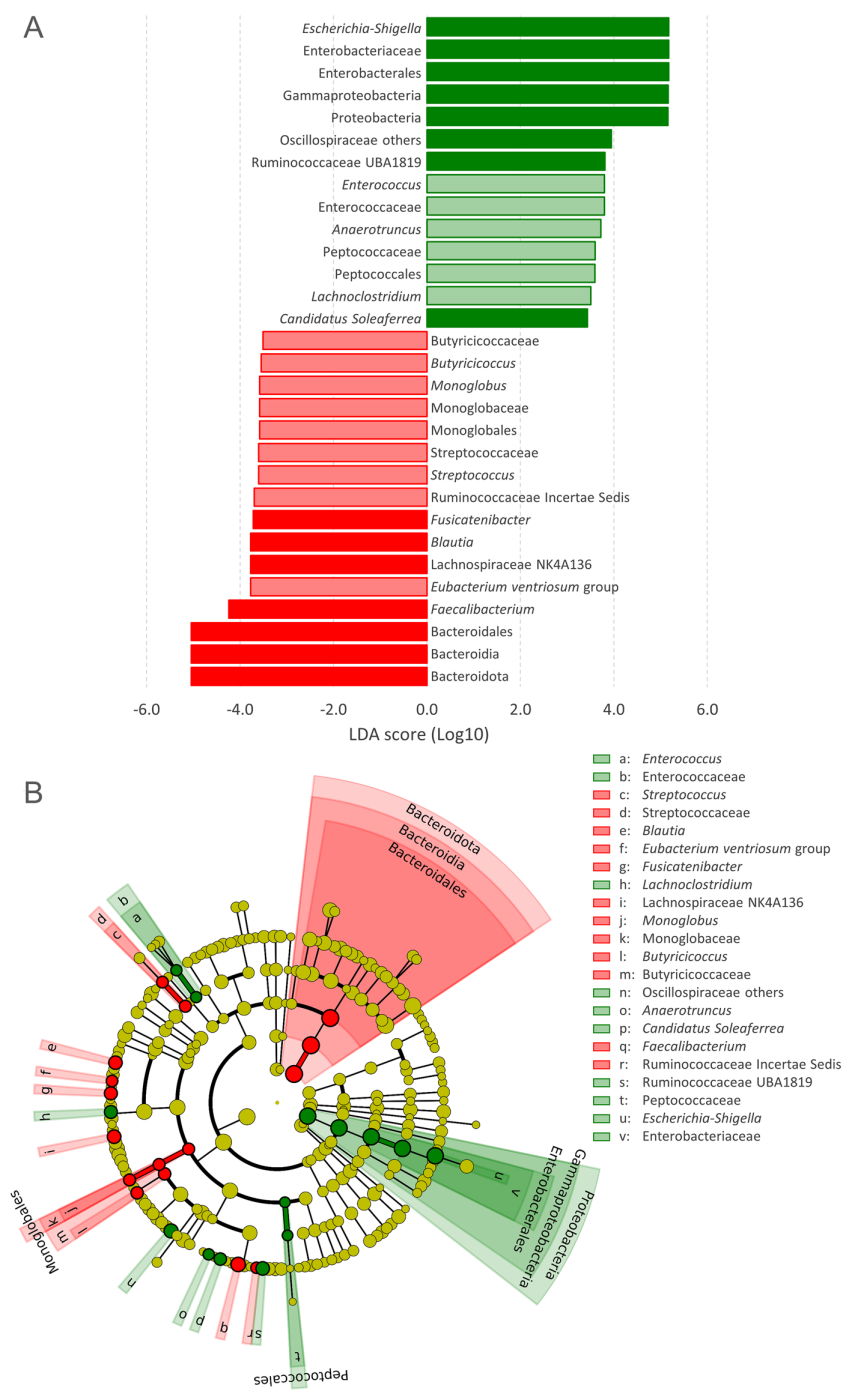
Effect of mucin enrichment on faecal microbiota. The taxonomic biomarkers characterizing FS and EC according to LEfSe are colored in red and green, respectively. (**A**) Logarithmic LDA scores of the taxa exhibiting significant differential abundance (P < 0.05, logarithmic LDA score ≥ 2.0) between FS and EC; taxa that were > 1% in at least one sample are reported with bars in dark shades. (**B**) Cladogram visualization of the taxonomic biomarkers. The figure was created with the software LEfSe, Galaxy version 1.0, available at https://huttenhower.sph.harvard.edu/galaxy/.

The microbiota of FS samples encompassed 125 of the 150 identified OTUs. It was dominated by Bacteroidota (59.5%), with remarkably high amounts of *Bacteroides* (39.3%) and Prevotellaceae (8.4%), followed by Firmicutes (35.3%), especially Lachnospiraceae (15.4%), and Proteobacteria (2.2%; Fig. 2). The microbiota of EC samples encompassed 101 OTUs, 25 of which became detectable because of the enrichment steps, as they lay below the limit of detection in FS samples. EC were dominated by Bacteroidota (39.3%) and Proteobacteria (30.8%). *Escherichia-Shigella* (with Enterobacteriaceae, Enterobacteriales, Gammaproteobacteria, and Proteobacteria) presented a significant positive differential abundance in EC compared to FS (Fig. 3) and represented one of the dominant genera in EC samples (mean = 30.5%), with remarkably high abundance in sample EC-5 (56.7%). Several taxa of Firmicutes, such as *Enterococcus* and many Clostridia (e.g., Oscillospiraceae, Peptococcaceae, Ruminococcaceae UBA1819, *Soleaferrea*, *Lachnoclostridium*, and *Anaerotruncus*), presented a significant increase in EC samples, but generally remained below a mean abundance of 1%.

A number of taxa were not detected in any FS sample but appeared after the enrichment: *Eggerthella* occurred in 4 EC samples; *Clostridium innocuum*, *Enterococcus durans*, *Erysipelatoclostridium*, and *Soleaferrea* in 3; *Angelakisiella* and *Eubacterium nodatum* in 2 EC samples. These taxa were generally a minor portion of the microbiota (< 1%), except for *Clostridium innocuum* that reached the 7.3% in EC-2. *Enterorhabdus*, *Gordonibacter*, *Dielma*, *Enterococcus faecalis* and other *Enterococcus* spp., *Catabacter, Ruminiclostridium*, Lachnospiraceae FCS020 group, *Hydrogenoanaerobacterium*, *Caproiciproducens*, Ruminococcaceae DTU089, *Peptostreptococcus, Peptoniphilus, Tissierella, Sedimentibacter, Citrobacter*, and *Cloacibacillus* were detected in a single EC sample, generally in very low concentration, except for *Dielma* (1%) and *Cloacibacillus* (1.5%).

Bacteroidota, Bacteroidia, and Bacteroidales, significantly decreased as consequence of mucin enrichment, albeit the genus *Bacteroides* remained the most abundant in 4 out of 5 EC samples. The genus *Streptococcus* also diminished, leading to significantly lower abundances also of Streptococcaceae. Numerous taxa of Clostridia decreased during the enrichment steps, such as *Eubacterium ventriosum*, the genera *Faecalibacterium, Fusicatenibacter, Blautia, Monoglobus* (plus Monoglobaceae and Monoglobales), and *Butyricicoccus* (and Butyricicoccaceae). Furthermore, the abundance of a clade of Lachnospiraceae (NK4A136) and Ruminococcaceae *incertae sedis* significantly diminished. Other taxa detected only in some FS samples decreased below the limit of detection in the EC samples, such as Coriobacteriales *incertae sedis,* other Eggerthellaceae, *Paraprevotella*, Defluviitaleaceae_UCG-011, *Eubacterium siraeum* group, *Veillonella*, *Butyricimonas*, Erysipelotrichaceae_UCG-003, *Turicibacter*, *Coprococcus*, *Lachnospira*, *Roseburia*, Anaerovoracaceae Family XIII UCG-001, and *Haemophilus*.

**Figure 4:**
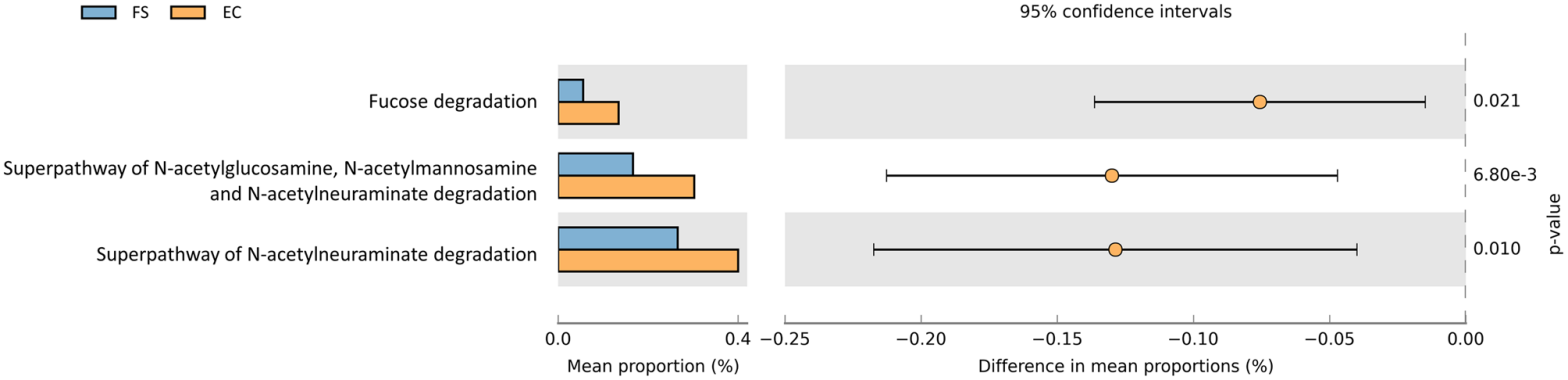
Pathways related to mucin degradation reconstructed from 16S rRNA profile with PICRUSt2 and presenting a significant (P < 0.05) differential abundance between FS and EC. The figure was created with STAMP v2.1.3, available at https://beikolab.cs.dal.ca/software/STAMP.

The functions of the fecal cultures’ metagenomes were predicted with PICRUSt2. A significant increase (t-test, p < 0.05) of pathways related to mucin metabolism was detected, i.e. fucose degradation, superpathway of N-acetylneuraminate degradation, and superpathway of GlcNAc, N-acetylmannosamine, and N-acetylneuraminate degradation (Fig. 4). In particular, genes encoding N-acetylglucosamine kinase (EC:2.7.1.59), N-acetylglucosamine-6-phosphate deacetylase (EC:3.5.1.25), N-acetylneuraminate epimerase (EC:5.1.3.24), N-acylglucosamine-6-phosphate 2-epimerase (EC:5.1.3.9), N-acylmannosamine kinase (EC:2.7.1.60) showed a statistically significant increase in the enriched cultures.

## Discussion

This study aimed to identify and isolate the intestinal bacteria that can have a role in mucin degradation, utilizing three steps of enrichment where the intestinal microbiota of healthy subjects was inoculated in a medium containing mucin as the sole carbon and nitrogen source. Mucin can support the growth of gut microbes with different metabolic capabilities. Both saccharolytic and proteolytic activities are involved in its breakdown, providing the bacterial community with carbon and nitrogen supply and sustaining growth of both sugar and amino acid fermenters. Based on the increase of genes and pathways involved in mucin degradation, the cultures successfully selected bacteria performing mucin breakdown and fermentation.

The 16S rRNA gene profiling highlighted the taxa that proliferated in mucin, likely because of cooperation and crossfeeding. Proteobacteria and taxa related to Enterobacteriaceae encompassed the main biomarkers of growth, in agreement with their major role as protein degraders in gut microbiota. As opportunistic commensals, they may persist in the gut contributing to the mucus homeostasis without interacting with epithelial cells or they may degrade mucins, reach the epithelium, and initiate disease^12–14^. The high affinity of Enterobacteriaceae for mucins confirms the possibility that they may get in contact with the intestinal epithelial barrier and exert this pathogenic action. In fact, these bacteria exhibit an intrinsic resistance to the oxygen released from the host tissue and may concur to disrupt the integrity of the epithelium, promoting a leaky gut and triggering systemic inflammatory response^15–17^.

Enterobacteriaceae, Peptococcales/Peptococcaceae, a variety of Clostridia (such as Oscillospiraceae, *Lachnoclostridium*, and *Anaerotruncus*), and the Coriobacteriale *Eggerthella,* are protein degraders that may have taken part to the breakdown of protein backbone of mucin^18^. The genus *Anaerotruncus* includes species (*Anaerotruncus colihominis* and *Anaerotruncus massiliensis*) that ferment some amino acids and also a small set of carbohydrates, including GlcNAc, and may have participated to the degradation of both the protein and saccharidic portions of mucins^19,20^. *Holdemania*, a Firmicute belonging to the family Erysipelotrichaceae, and Enterococcaceae (Lactobacillales) are saccharolytic bacteria, thus their contribution to mucin degradation seems limited to the carbohydrate chains. Several other taxa, which are still poorly characterized at the physiological level, emerged as participating in mucin catabolism, for some species representing one of the first sources of functional information. Interestingly, the list of bacteria that got enriched in EC includes taxa that are associated to colorectal adenoma and cancer (e.g. *Lachnoclostridium*) and are emerging as uncommon human pathogens (*Eggerthella* and some Enterococcaceae)^21–23^.

Even though the 16S rRNA gene profiling disclosed a complex population of bacteria taking advantage of mucin, only three species of strict anaerobic bacteria (*C. disporicum*, *C. tertium*, and *P. benzoelyticum*) were isolated from the cultures enriched with 5 different microbiotas. *C. disporicum* is a saccharolytic bacterium that presents epimerase activity resulting in the production of ursodeoxycholic acid^24,25^. *C. tertium* is an uncommon pathogen, albeit associated with cases of severe diseases, mostly in immunocompromised and neutropenic patients^26^. *P. benzoelyticum* can ferment some amino acids, including threonine, a major component of mucin backbone^27^. Interestingly, the isolates of *C. disporicum* and *C. tertium* are all designated as *Clostridium *sensu strictu 1 according to SILVA database. The 16S rRNA gene profiling revealed that the OTU *Clostridium *sensu strictu 1 increased in all the samples where it was present, although it did not reach a significant differential abundance, and was composed of four ASVs contributing to FS and EC separation along PCo2 in the plot of Unweighted UniFrac beta-diversity.

Albeit *Clostridium* species harbor few glycosyl hydrolases (GH) involved in mucin degradation and, for instance, genome of *C. sporogenes,* a closely related species to *C. tertium*, has no mucin-related GHs, based on preliminary genomic analysis (data not shown), the genome of *C. tertium* WC 0709 has at least one exo-α-sialidase (EC 3.2.1.18) belonging to the GH33 family, involved in the cleavage of the terminal sialic acid, and a GH3 family β-hexosaminidase (EC 3.2.1.52) that can release GalNac. On the other side *P. bifermentans*, closely related to *P. benzoelyticum*, harbors only the galactosidases GH2 and GH20. Generally, commensal clostridia do not appear to harbor GH33 or other mucin-related GHs, whereas two pathogenic clostridia degrade mucin glycans: *C. perfingens* and *C. septicum*^28^*.* The capability of *C. disporicum*, *C. tertium*, and *P. benzoelyticum* to dismantle complex mucin glycans deserves deeper investigations and opens new perspectives on the relationship of these species with the host, that can go beyond the commensalism. Incidentally, *C. tertium* is described as an uncommon pathogen associated with various cases of severe diseases^26,29^. The glycans of intestinal mucins, enriched in diverse monosaccharides with different positional and stereochemical linkages and branching, are hydrolyzed by a number of diverse sialidases, hexosaminidases, galactosidases, and fucosidases. The genetic and functional characterization of the enzymes that in *C. disporicum*, *C. tertium*, and *P. benzoelyticum* play key roles in disassembling mucin oligosaccharides is a great challenge.

In the gut, a complex community of saccharolytic and proteolytic bacteria is expected to participate to mucin breakdown, with linkage-specific glycosidases and proteases of primary degraders providing other bacteria with simpler oligosaccharides and peptides, and eventually with their monomeric moieties. It is plausible that several taxa detected by the 16S rRNA gene analysis could have grown on mucins only when co-cultured within a bacterial community where each species contributed with different hydrolytic activities, while they could not grow in pure cultures where they did not benefit from crossfeeding relationships. Only *C. disporicum*, *C. tertium*, and *P. benzoelyticum* gained carbon, energy, and nitrogen from mucin in pure cultures. The isolates of these species deserve deeper investigation, including the genome sequencing and physiology studies, aiming to unveil the key functions that enabled growth on mucin, in particular their set of glycosyl hydrolases and proteases and their amino acid biosynthetic capability.

To date, *Bacteroides* and *Akkermansia muciniphila* have been recognized as main mucin degraders, with novel insights on endo-acting O-glycanases involved in the first steps of glycan breakdown^30^. In this study, the relative abundance of *Bacteroides* did not increase significantly, even if some ASVs of *Bacteroides* heavily contributed to separate samples along both PCo1 or PCo2 in the Weighted UniFrac analysis, confirming the presence of some species greatly associated to mucin degradation. It is plausible that different populations of *Bacteroides*, which could not be discriminated into species by the partial 16S rRNA gene sequencing, behaved differently with respect to mucin breakdown, exerting contrary effects and balancing the counts of the genus. Indeed, a wide number of ASVs attributed to *Bacteroides* presented opposite contributions along the principal coordinates, in agreement with the fact that the genus *Bacteroides* comprises tens of species that supply different and complementary functions to the intestinal community^31^.

One of the key members of the colonic mucus-associated microbiota is *A. muciniphila*. It releases monosaccharides and amino acids during mucin degradation, providing nutrients to other bacteria of the gut, and exerts beneficial effects in various metabolic disorders, being generally negatively correlated with inflammation and metabolic disorders^32^. In this study, a sole ASV ascribed to *Akkermansia* was detected in 3 out of 5 set of samples. In subject 1, it was remarkably abundant in the founding microbiota (8.7%) but was negligible (0.2%) after the enrichment steps. In samples 3 and 4, *Akkermansia* was initially < 0.6% and increased by one magnitude over the enrichment. It is plausible that in sample 1 the high initial load of *Akkermansia* prevented a net increase of the abundance, that was further squeezed by major increase in some other groups.

The absence of *Bacteroides* and *Akkermansia* also within the isolates is noteworthy. These bacteria likely encountered some substrate limitation that hampered growth. In fact, the medium did not contain nitrogen sources other than mucin in order to be as much stringent as possible. Thus, all the amino acids had to be obtained from the protein backbone of mucin, and this might have been too challenging, in the absence of a cooperative crossfeeding. The composition of the medium and the choice of the pH are major drivers of microbial selection and a mucin-based medium with different composition would likely result in the isolation of different bacterial species. Another drawback of the present study was the use of mucin from porcine stomach, that mainly consists of MUC5AC and MUC6 mucins and differs in purity and glycosylation profile from the colonic one MUC2, but is a cheap and easily available substrate, commonly utilized to culture mucin degraders^5,33–35^.

This study shed light on the bacterial taxa thriving in cultures of human microbiota with mucin as primary substrate for growth. It provided the first information on *C. disporicum*, *C. tertium*, and *P. benzoelyticum* as bacteria able to grow in pure cultures utilizing mucin as sole carbon and nitrogen source, complementing the recognized role of *Bacteroides* and *Akkermansia* in mucin degradation and turnover. Sequencing of the 10 strains is in progress, as well as the formulation of a new mucin-based medium allowing a faster growth with higher yields, in order to better address physiology and metabolism studies. Proteobacteria, including Enterobacteriaceae and *E. coli*, are among the microbial group that most benefit from mucin as substrate, increasing the risk of the flourishing of opportunistic pathogens or bacteria associated to gut inflammation. Furthermore, metataxonomic analysis identified *Lachnoclostridium*, *Eggerthella*, *Anaerotruncus*, *Enterococcaceae*, and *Peptococcaceae* as taxa that took advantage, likely in a cross-feeding relationship, in the mucin-based medium. The limited number of isolated species able to grow on mucin in pure culture confirms that the degradation of these glycoproteins in the gut results from cooperation and cross-feeding among several species exhibiting different metabolic capabilities.

## Materials and methods

### Chemicals and media

All the chemicals were purchased from Sigma-Aldrich (Merck KGaA, Darmstadt, Germany), unless otherwise stated. Mucin was purified from porcine stomach type II mucin (Sigma-Aldrich), according to the protocol by Miller and Hoskins^36^. Briefly, mucin was precipitated with ice-cold ethanol at the concentration of 60% (v/v) from a clarified solution, obtained by centrifugation (10,000×*g* for 10 min at 4 °C) of a 25 g/L mucin suspension in 0.1 M NaCl containing 0.02 M phosphate buffer, pH 7.6. The precipitate was dissolved in 0.1 M NaCl, re-precipitated with ethanol (60% v/v), and dissolved in water. The mucin solution was dialyzed with (14 kDa cut-off) against water and finally freeze dried. According to the producer, mucin had a molecular mass of 4000–5500 kDa, with a carbohydrate portion of 80–84%, and a protein portion of 16–20%. Proline, serine, and threonine residues in the peptide backbone comprise approx. 43% of the amino acid composition.

MM medium contained 3.0 g/L purified mucin, 2.0 g/L KH_2_PO_4_, 4.5 g/L NaCl, 0.5 g/L MgSO_4_·7H_2_O, 0.045 g/L CaCl_2_·2H_2_O, 0.005 g/L FeSO_4_·7H_2_O, 0.01 g/L hemin, 0.05 g/L bile salts (Oxgall, BD Difco, Sparks, MD, USA), 0.6 mg/L resazurin, 2.0 mL/L minerals solution (0.5 g/L EDTA, 0.010 g/L ZnSO_4_·7H_2_O, 0.003 g/L MnCl_2_·7H_2_O, 0.03 g/L H_3_BO_3_, 0.02 g/L CoCl_2_·6H_2_0, 0.001 g/L CuCl_2_·2H_2_O, 0.002 g/L NiCl_2_·6H_2_O, and 0.003 g/L NaMoO_4_·2H_2_O), 1.4 mL/L vitamins solution (1.0 g/L menadione, 2.0 g/L biotin, 2.0 g/L calcium pantothenate, 10 g/L nicotinamide, 0.5 g/L cyanocobalamin, 0.5 g/L folic acid, 4 g/L thiamine, and 5 g/L PABA), and 40 mL/L reducing solution (12.5 g/L l-cysteine·HCl and 80 g/L NaHCO_3_). The basal medium was autoclaved at 121 °C for 20 min and supplemented with filter sterilized (0.22 µm) minerals, vitamins, and reducing solutions. Soft-agar MM plates, containing 8 g/L agar (BD Difco), were used to count, and purify mucin degraders. MacConkey agar plates (BD Difco) were utilized to count *Escherichia coli* and Enterobacteriaceae and to discriminate them from strictly anaerobic isolates.

### Enrichment cultures

This study was carried out under the recommendations of the protocol approved with ref. no. 125-15 by the local research ethics committee (Comitato Etico Provinciale, Azienda Policlinico di Modena, Italy), with written informed consent from all subjects following the Declaration of Helsinki. Five healthy adults (3 men and 2 women, aged 25–50 years) who had not taken prebiotics and/or probiotics in the previous 2 weeks or antibiotics for at least 3 months were enrolled for feces collection. Fecal samples were collected fresh, sealed in anaerobic plastic bags (AnaeroGen, Oxoid, Basingstoke, UK), transferred in an anaerobic cabinet (Concept Plus, Ruskinn Technology, Ltd., Bridgend, UK) with an atmosphere of 85% N_2_, 10% CO_2_, and 5% H_2_, and homogenized (10%, w/v) in sterile MM.

The fecal slurries (FS) were inoculated with a syringe (10% v/v) into butyl-rubber stoppered bottles containing 50 mL of sterile anaerobic MM. After an incubation of 72 h at 37 °C, these cultures were utilized to seed (10% v/v) the next passage in MM, repeated to accomplish three enrichment steps. Once grown, the third enrichment culture (EC) was utilized for isolation of mucinolytic bacteria. The viable counts of anaerobic mucinolytic bacteria and Enterobacteriaceae in EC samples were determined in triplicate onto agar plates of MM and MacConkey, respectively. For both the media, one-way ANOVA followed by Tukey’s *post-hoc* analysis was utilized to compare the counts of the different subjects. Paired samples t-test was utilized to compare MM and MacConkey counts.

### Isolation of anaerobic mucin degraders

All the steps of the isolation and purification procedure were carried out in the anaerobic cabinet. The EC was serially diluted (1:10) in anaerobic MM, then 1 mL of each dilution was poured into plates and covered with 20 mL of melted soft-agar MM. The plates were allowed to cool and incubated in anaerobiosis at 37 °C for 72–96 h. Inclusion colonies were picked and checked for aerobic growth at 37 °C in MacConkey agar plates. Colonies unable to grow in aerobiosis were further purified at least three times by serial dilution and isolation in soft-agar MM.

Approximately 10 pure clones per sample were fingerprinted by RAPD-PCR according to Quartieri et al.^37^ and clustered into biotypes with a similarity level of 75% using the Pearson correlation coefficient. Single biotypes from each sample were taxonomically characterized by partial sequencing of the 16S rRNA gene (Eurofins Genomics, Ebersberg, Germany), utilizing primers targeting the V1–V4 regions^38^. Sequences were compared to the NCBI refseq_rna database with the alignment tools BLAST and MUSCLE (https://www.ncbi.nlm.nih.gov/; https://www.ebi.ac.uk/Tools/msa/muscle/).

### 16S rRNA gene profiling

Total DNA was extracted from 2 mL of FS and EC samples with the QiAmp PowerFecal DNA kit (Qiagen, Hilden, Germany), according to the manufacturer’s protocol. The DNA was normalized to 5 ng/µL after quantification with a Qubit 3.0 fluorimeter (Thermo Fisher Scientific, Waltham, MA, USA).

Partial 16S rRNA gene sequences were amplified using Probio_Uni/Probio_Rev primers, which targeted the V3 region of the 16S rRNA gene. Amplicons were sequenced using a MiSeq platform (Illumina Inc., San Diego, CA, USA) according to Milani et al.^39^. Raw sequences were analyzed with the QIIME 2.0 pipeline, version 2019.10^40^, with appropriate plugins for trimming (CUTADAPT) and denoising (DADA2) into amplicon sequence variants (ASVs)^41,42^. Taxonomy assignment was carried out with the feature classifier VSEARCH^43^, with SILVA SSU database release 138 (https://www.arb-silva.de/download/arb-files/) as reference and the the similarity threshold set at 0.97. The appropriate QIIME2 plugins were utilized to compute the alpha- (observed taxa, Chao1, Shannon, and Pielou’s evenness) and beta-diversity (Unweighted Normalized UniFrac, and Weighted Normalized UniFrac) and to compare them within and between FS and EC samples (i.e., the Kruskal–Wallis test for alpha diversity; ANOSIM and PERMANOVA for beta diversity). Differences were considered significant for P < 0.05. Principal Coordinate Analysis (PCoA) was computed with QIIME2, based on the beta-diversity distance matrices.

Linear discriminant analysis Effect Size (LEfSe, http://huttenhower.sph.harvard.edu/galaxy) algorithm was utilized to discover distinctive taxonomic features characterizing FS and EC samples^44^. Taxa presenting a significant differential abundance (P < 0.05) and logarithmic LDA (linear discriminant analysis) score > 2 were considered as microbial biomarkers of FS or EC samples.

The dataset was analyzed with PICRUSt2 to predict metagenomes’ functions and investigate the presence of any difference between the FS and EC samples in terms of enzymes and pathways related to mucin metabolism^45^. The tool predicted the enzymes and the relative MetaCyc pathways abundances^46^. STAMP was used to visualize the results and conduct statistical analysis^47^.

The 16S rRNA gene sequences datasets generated and analyzed during the current study are available in the NCBI repository with the BioProject ID: PRJNA649741 (https://www.ncbi.nlm.nih.gov/).

## Supplementary Information

Supplementary Information 1.

Supplementary Information 2.
