# Supplementary material for: Identification of mucin degraders of the human gut microbiota
Source: Sci Rep. 2021 May 27;11:11094. doi: 10.1038/s41598-021-90553-4 (PMC8159939; doi:10.1038/s41598-021-90553-4)
Supplement: Supplementary file 1 — Supplementary Information 1. [file 41598_2021_90553_MOESM1_ESM.pdf]

Identification of mucin degraders of the human gut microbiota

Stefano Raimondi[1†], Eliana Musmeci[1†], Francesco Candeliere[1], Alberto Amaretti[1,2], and Maddalena Rossi[1,2,*]
[1]Department of Life Sciences, University of Modena and Reggio Emilia, 41125 Modena, Italy
[2]Biogest-Siteia, University of Modena and Reggio Emilia, 42122 Reggio Emilia, Italy
[†] The authors contributed equally to this work
*corresponding author: maddalena.rossi@unimore.it

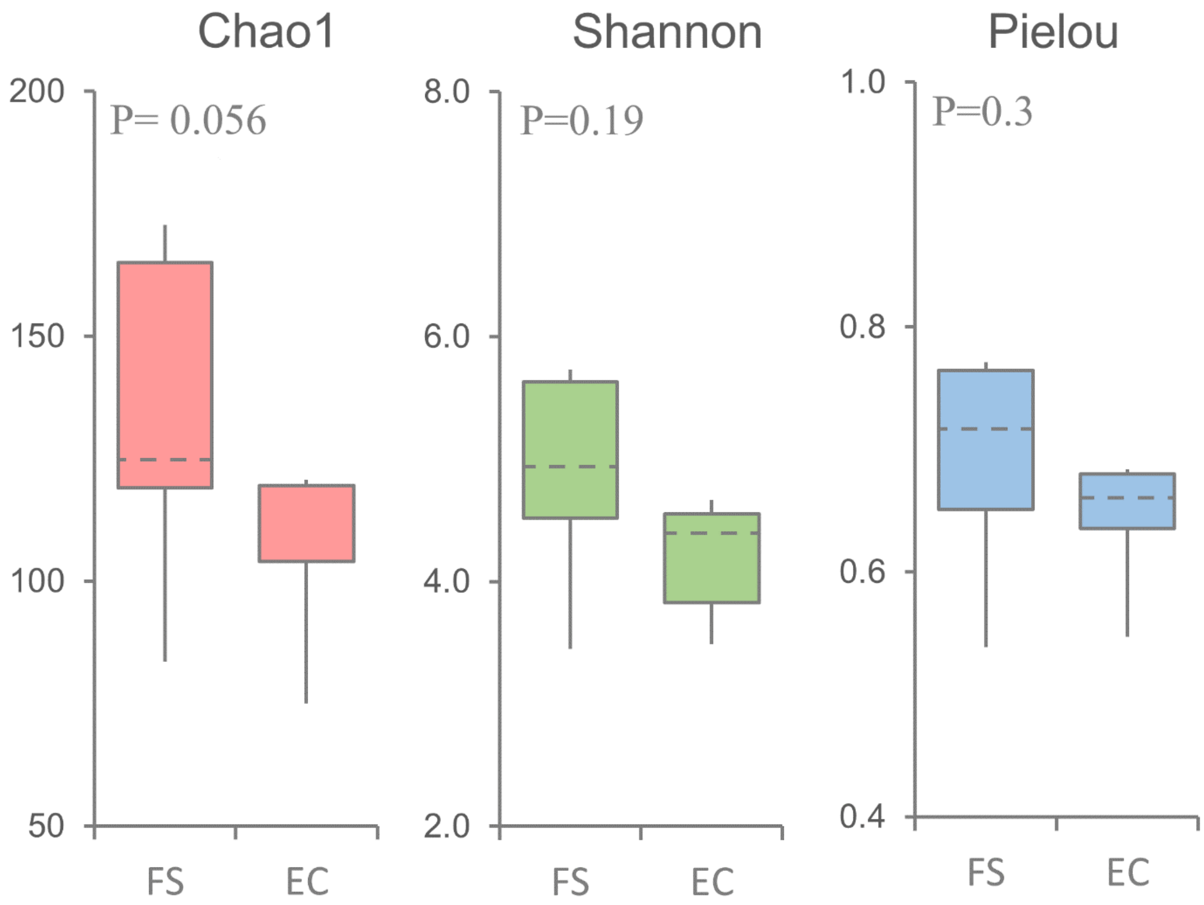

Supplementary Figure 1. Alpha diversity metrics, comprising Chao1, Shannon, and Pielou indexes, of the microbiota in FS and EC samples. The median (dashed line), the 25th and 75th percentiles (colored box), the 10th and 90th percentiles (whiskers), are indicated. Paired FS and EC samples were compared with Kruskal-Wallis test, considering significant difference for $P < 0.05$.

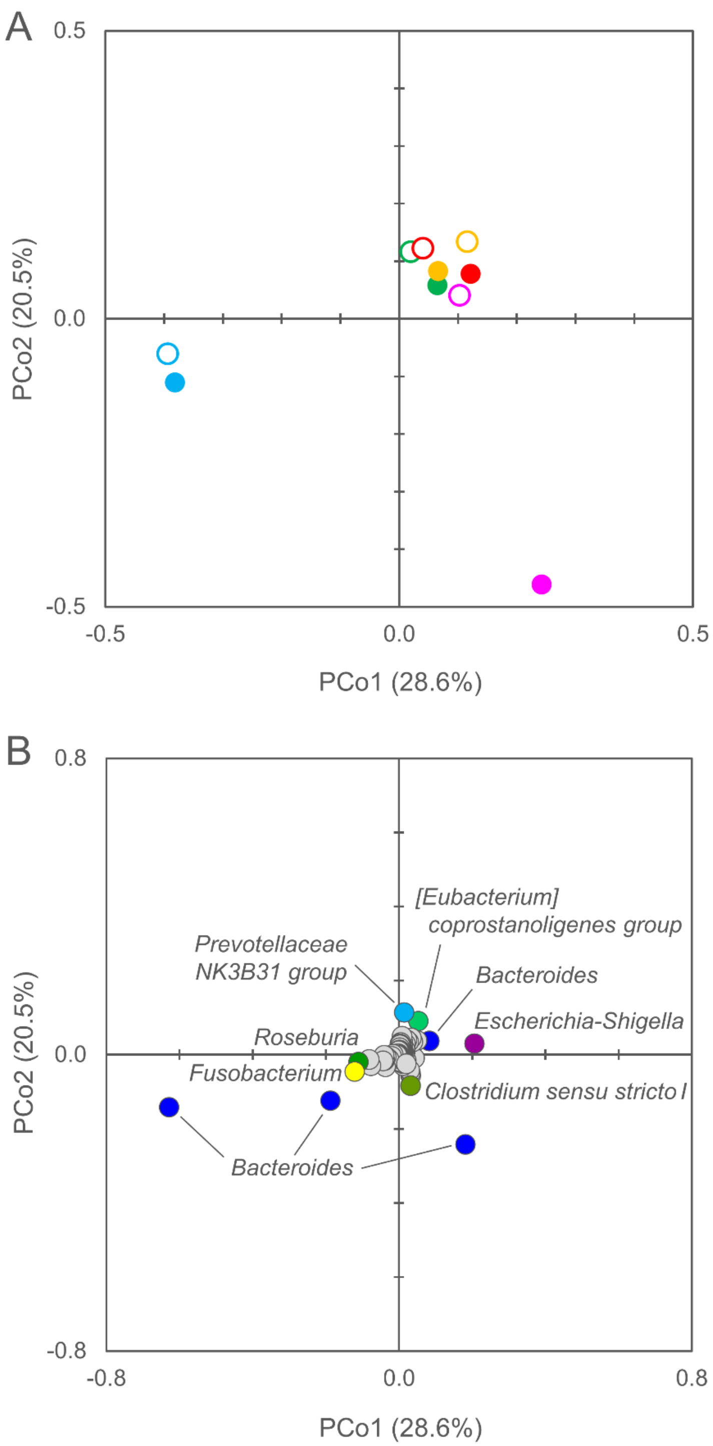

Supplementary Figure 2 Beta diversity analysis of the microbiota in FS and EC samples. Panel A, PCo1-PCo2 visualization of distances computed with Unweighted Unifrac. Symbols: FS, empty circle; EC, full circle; different colors correspond to different subjects (1, fuchsia; 2, cyan; 3, green; 4, red; 5, yellow). Panel B, PCo1-PCo2 visualization of the contribution of each ASV. The 10 ASVs exerting the highest effect on PCo1 are labelled and colored according to their phylum (Firmicutes, green; Bacteroidetes, blue; Preteobacteria, purple; Fusobacteria, yellow).
